# Molecular epidemiology of hepatitis C virus in Cambodia during 2016–2017

**DOI:** 10.1038/s41598-019-43785-4

**Published:** 2019-05-13

**Authors:** Janin Nouhin, Momoko Iwamoto, Sophearot Prak, Jean-Philippe Dousset, Kerya Phon, Seiha Heng, Alexandra Kerleguer, Mickaël Le Paih, Philippe Dussart, David Maman, François Rouet

**Affiliations:** 1 Virology Unit, Institut Pasteur du Cambodge, Institut Pasteur International Network, Phnom Penh, Cambodia; 2 Epicentre, Paris, France; 3 Médecins Sans Frontières – France, Phnom Penh, Cambodia; 4 Medical Laboratory, Institut Pasteur du Cambodge, Institut Pasteur International Network, Phnom Penh, Cambodia

**Keywords:** Epidemiology, Genotype, Data processing

## Abstract

In Cambodia, little epidemiological data of hepatitis C virus (HCV) is available. All previous studies were limited to only small or specific populations. In the present study, we performed a characterization of HCV genetic diversity based on demography, clinical data, and phylogenetic analysis of HCV non-structural 5B (NS5B) sequences belonging to a large cohort of patients (n = 3,133) coming from majority part of Cambodia between September 2016 and December 2017. The phylogenetic analysis revealed that HCV genotype 1 and 6 were the most predominant and sharing equal proportions (46%). The remaining genotypes were genotype 2 (4.3%) and unclassified variants (3.6%). Among genotype 1, subtype 1b was the most prevalent subtype accounting for 94%. Within genotype 6, we observed a high degree of diversity and the most common viral subtypes were 6e (44%) and 6r (23%). This characteristic points to the longstanding history of HCV in Cambodia. Geographic specificity of viral genotype was not observed. Risks of HCV infection were mainly associated with experience of an invasive medical procedure (64.7%), having partner with HCV (19.5%), and blood transfusion (9.9%). In addition, all of these factors were comparable among different HCV genotypes. All these features define the specificity of HCV epidemiology in Cambodia.

## Introduction

Hepatitis C virus (HCV), one of the causative agents of chronic liver disease, remains a global public health issue despite the availability of direct-acting antivirals (DAA). In 2015, the World Health Organization (WHO) estimated 71 million people worldwide were living with chronic hepatitis C and about 10 million from Southeast Asia^1^. HCV is a blood borne virus that is commonly transmitted through blood exposure including blood transfusion, sharing of drug injecting devices and reuse of contaminated medical equipment, in particular, syringes and needles. Exhibiting a high degree of genetic diversity^2^, HCV is classified into eight genotypes (1–8)^3,4^ and subdivided into 87 subtypes named in alphabetical order (1a – 1n, 2a – 2 u, 3a – 3k, 4a – 4w, 5a, 6a – 6xf, 7a – 7b, and 8a)^4,5^.

An understanding of HCV molecular epidemiology is important for surveillance of transmission dynamics and leads to an appropriate public health response. In the era of DAAs, knowledge of HCV genetic diversity may also be useful for the treatment and management of HCV-infected patients, particularly in case of virological failure. The majority of described hepatitis C infection and treatment outcome is associated with HCV genotype 1, which is globally distributed and well conserved^6,7^. In contrast, high diversity HCV lineages are observed in high endemic areas. For instance, in West Africa, endemic strains belonging to genotypes 1, 2, and 5 are highly prevalent^8–10^. Regional patterns of endemic diversity have been described for genotype 3 in the Indian subcontinent^11^, genotype 4 in North Africa and the Middle East^6^, genotype 5 in West^9,10^ and South Africa^12^, and genotype 6 in China and Southeast Asia^6,13^.

In Cambodia, a Southeast Asian country, data of HCV prevalence and molecular epidemiology are poorly documented. Previous studies were performed on small sample sizes of specific populations: blood donors^14^, human immunodeficiency virus (HIV) and HCV co-infected adults^15–17^, Cambodian immigrants in foreign countries^18^, and children^19^ – and with different age categories^19,20^. Consequently, an accurate prevalence of HCV in general population is unknown. The prevalence of HCV ranges between 2.8% and 14.7% in rural areas depending on study sites^14,19–21^ and between 5.5% and 10.4% among people with HIV in hospital-based programs in Phnom Penh, the capital city of Cambodia^15–17^. In terms of HCV genotype, there is a remarkable scarcity of data for this Southeast Asian country, compared to others in the region. Available sequences from previous studies revealed co-circulation of HCV genotype 1 and 6^16,18,22^.

In the present study, we report epidemiology of HCV genotypes in Cambodia based on a large number of data obtained from a Ministry of Health (MoH)-integrated HCV program.

## Results

### Study population

Table 1 describes sociodemographic and clinical characteristics of the 3,133 patients included in the study. The average age and standard deviation (SD) of the study population was 55.0 (SD, 11.2) years (range, 18–87) and 59% were female (Table 1). Forty-four percent (n = 1,374/3,133) of the patients were from Phnom Penh, the capital city of Cambodia and its vicinity (n = 1,006 from Phnom Penh, n = 368 from Kandal Province) and a greater proportion were from outside the capital (56%, n = 1,758/3,131). Nearly all patients were Cambodian (99.9%) and most (94.2%) were naïve to HCV treatment including interferon and ribavirin.

**Table 1:** Sociodemographic and clinical characteristics of patients.

Patient characteristics	All subjects (n = 3,133)	Genotype 1 (n = 1,444)	Genotype 2 (n = 134)	Genotype 6 (n = 1,442)	Non classified (n = 113)	p-values
**Demography**						
Age (years), mean (SD)	55.0 (11.2)	54.5 (11.6)	56.6 (11.6)	55.5 (10.9)	54.4 (10.0)	p < 0.05
Female	1,840 (59%)	863 (60%)	90 (67%)	814 (56%)	73 (65%)	p < 0.05
BMI (kg/m^2^), mean (SD)	23.7 (3.8)	23.6 (3.7)	23.9 (3.6)	23.7 (4.0)	24.2 (3.4)	p < 0.05
Cambodian Nationality, n (%)	3,130 (99.9%)	1,442 (99.9%)	134 (100.0%)	1,441 (99.9%)	113 (100.0%)	p = 0.89
Alcohol use (AUDIT ≥ 8)	112/3,115 (3.4%)	55/1,432 (3.8%)	8/134 (6.0%)	46/1,437 (3.2%)	3/113 (2.7%)	p = 0.34
**Place of residence**						
Phnom Penh Municipality	1,006	421	56	490	39	
Kandal Province	368	176	18	164	10	
Kampong Chhnang Province	305	191	12	88	14	
Battambang Province	211	89	8	106	8	
Banteay Meanchey Province	174	88	4	82	0	
Kampong Cham Province	156	61	5	83	7	
Kampot Province	136	55	6	69	6	
Takeo Province	124	65	5	50	4	
Siem Reap Province	123	42	1	75	5	
Kampong Speu Province	98	60	2	33	3	
Pursat Province	73	29	3	38	3	
Preah Sihanouk Province	71	35	2	30	4	
Prey Veng Province	62	36	1	22	3	
Kampong Thom Province	45	27	1	17	0	
Tboung Khmum Province	36	11	2	23	0	
Pailin Province	28	9	0	19	0	
Kratie Province	25	16	1	8	0	
Svay Rieng Province	24	7	6	7	4	
Koh Kong Province	17	7	1	9	0	
Oddar Meanchey Province	13	2	0	11	0	
Preah Vihear	13	4	0	7	2	
Mondulkiri Province	11	2	0	9	0	
Ratanakiri Province	7	4	0	2	0	
Stung Treng Province	4	3	0	0	1	
Kep Province	2	2	0	0	0	
Unknown	1	1	0	0	0	
**Risk factors for infection, n (%)**						
Invasive medical procedures	2,021/3,124 (64.7%)	910/1,439 (63.2%)	81/134 (60.4%)	956/1,438 (66.5%)	74/113 (65.5%)	p = 0.22
Blood transfusion	307/3,107 (9.9%)	145/1,431 (10.1%)	6/132 (4.5%)	144/1,431 (10.0%)	12/113 (10.6%)	p = 0.22
Partner with HCV	461/2,367 (19.5%)	210/1,109 (18.9%)	23/96 (23.9%)	210/1,074 (19.6%)	18/88 (20.5%)	p = 0.68
Healthcare worker	220/3,119 (7.1%)	89/1,437 (6.2%)	13/134 (9.7%)	107/1,436 (7.5%)	11/112 (9.8%)	p = 0.19
Imprisonment	40/3,108 (1.3%)	22/1,429 (1.5%)	1/132 (0.8%)	16/1,435 (1.1%)	1/112 (0.9%)	p = 0.69
FEW, MSM, or TG	27/3,119 (0.9%)	16/1,434 (1.1%)	1/134 (0.7%)	10/1,438 (0.7%)	0/113 (0.0%)	p = 0.47
History of drug use	18/3,120 (0.6%)	12/1,435 (0.8%)	1/134 (0.7%)	5/1,438 (0.3%)	0/113 (0.0%)	p = 0.29
**Previous treatment history, n (%)**						
Naïve	2,951 (94.2%)	1,351 (93.6%)	126 (94.0%)	1,365 (94.7%)	109 (96.4%)	p = 0.51
IFN or pegIFN ± RBV	135 (4.3%)	74 (5.1%)	6 (4.5%)	52 (3.6%)	3 (2.6%)	
RBV monotherapy	25 (0.8%)	12 (0.8%)	1 (0.7%)	12 (0.8%)	0 (0.0%)	
DAAs	22 (0.7%)	7 (0.5%)	1 (0.7%)	13 (0.9%)	1 (0.9%)	
**Virology and fibrosis stage**						
Baseline HCV RNA Log_10_ (IU/mL), median (IQR)	6.1 (5.4–6.7)	5.9 (5.3–6.5)	6.1 (5.1–6.7)	6.3 (5.6–6.8)	6.4 (5.4–6.8)	p < 0.001
Baseline HCV RNA ≥ 800 000 (IU/mL), n (%)	1819 (58.1%)	736 (51.0%)	77 (57.5%)	935 (64.8%)	71 (62.8%)	p < 0.001
Fibroscan (kPa), median (IQR)	10.0 (6.4–17.6)	10.4 (6.6–20.0)	8.6 (5.8–15.6)	9.7 (6.4–17.0)	9.2 (5.9–17.0)	p < 0.05
**Fibrosis stage, n (%)**						
F0	338 (10.8%)	144/1420 (10.1%)	18/131 (13.7%)	157/1435 (10.9%)	19/113 (16.8%)	p = 0.06
F1	650 (21.0%)	284/1420 (20.0%)	28/131 (21.4%)	315/1,435 (21.9%)	23/113 (20.35%)	
F2	497 (15.9%)	222/1,420 (15.6%)	28/131 (21.4%)	231/1,435 (16.1%)	16/113 (14.1%)	
F3	579 (18.5%)	252/1420 (17.7%)	19/131 (14.5%)	286/1,435 (19.9%)	22/113 (19.5%)	
F4 (compensated cirrhosis)	928 (29.6%)	460/1,420 (32.4%)	31/131 (23.7%)	406/1,435 (28.3%)	31/113 (27.4%)	
F4 (decompensated)	107 (3.4%)	58/1,420 (4.1%)	7/131 (5.3%)	40/1,435 (2.8%)	2/113 (1.8%)	
**Laboratory findings, median (IQR)**						
Hemoglobin (g/dL)	12.9 (11.9–14.0)	12.9 (11.8–14.0)	12.8 (11.9–13.6)	12.9 (12.0–14.0)	12.8 (11.9–14.0)	p = 0.59
Platelet count (10^3^cells/μL)	165 (114–216)	161 (111–216)	164 (109–215)	167 (117–217)	171 (132–213)	p = 0.44
APRI	1.3 (0.7–2.7)	1.3 (0.7–2.7)	1.9 (0.7–3.3)	1.2 (0.6–2.6)	1.0 (0.6–2.3)	p = 0.10
MDRD eGFR (mL/min/1.73 m^2^)	64 (55–75)	64 (56–75)	66 (57–74)	65 (55–76)	66 (56–78)	p = 0.87
AST (IU/L)	67 (44–105)	68 (46–107)	80 (45–126)	66 (43–102)	53 (41–107)	p = 0.18
ALT (IU/L)	65 (41–101)	68 (41–99)	97 (48–136)	63 (41–99)	55 (39–116)	p = 0.07
**Comorbidities, n (%)**						
HIV co-infection	74/3128 (2.4%)	33/1439 (2.3%)	2/134 (1.5%)	37/1,442 (2.6%)	2/113 (1.7%)	p = 0.83
HBV co-infection	51/2548 (2.0%)	23/1172 (1.9%)	2/102 (1.9%)	24/1,181 (2.0%)	2/93 (2.2%)	p = 0.99
Hypertension	910/3127 (29.1%)	403/1438 (28.0%)	43/134 (32.1%)	424/1,442 (29.4%)	40/113 (35.4%)	p = 0.31
Diabetes (Type 1 or 2)	509 (16.3%)	223 (15.4%)	24 (17.9%)	239 (16.6%)	23 (20.3%)	p = 0.48

Notes: SD: standard deviation; IQR: interquartile range; FEW: female entertainment worker; MSM: men who have sex with men; TG: transgender; IFN: interferon; PegIFN: pegylated interferon; RBV: ribavirin; DAAs: direct-acting antivirals; APRI: aminotransferase-to-platelet ratio index; MDRD eGFR: modification of diet in renal disease study equation for estimating glomerular filtration rate; Laboratory findings are only for 1729 patients (825 genotype 1, 58 genotype 2, 787 genotype 6, 59 unassigned genotype) without missing laboratory results (hemoglobin, platelet, APRI, eGFR, AST, and ALT). P-values represent significance at alpha level of 0.05 for Chi-squared tests for categorical variables, ANOVA or Kruskal-Wallis test for continuous variables.

### HCV genotype and subtype distribution

**Figure 1:**
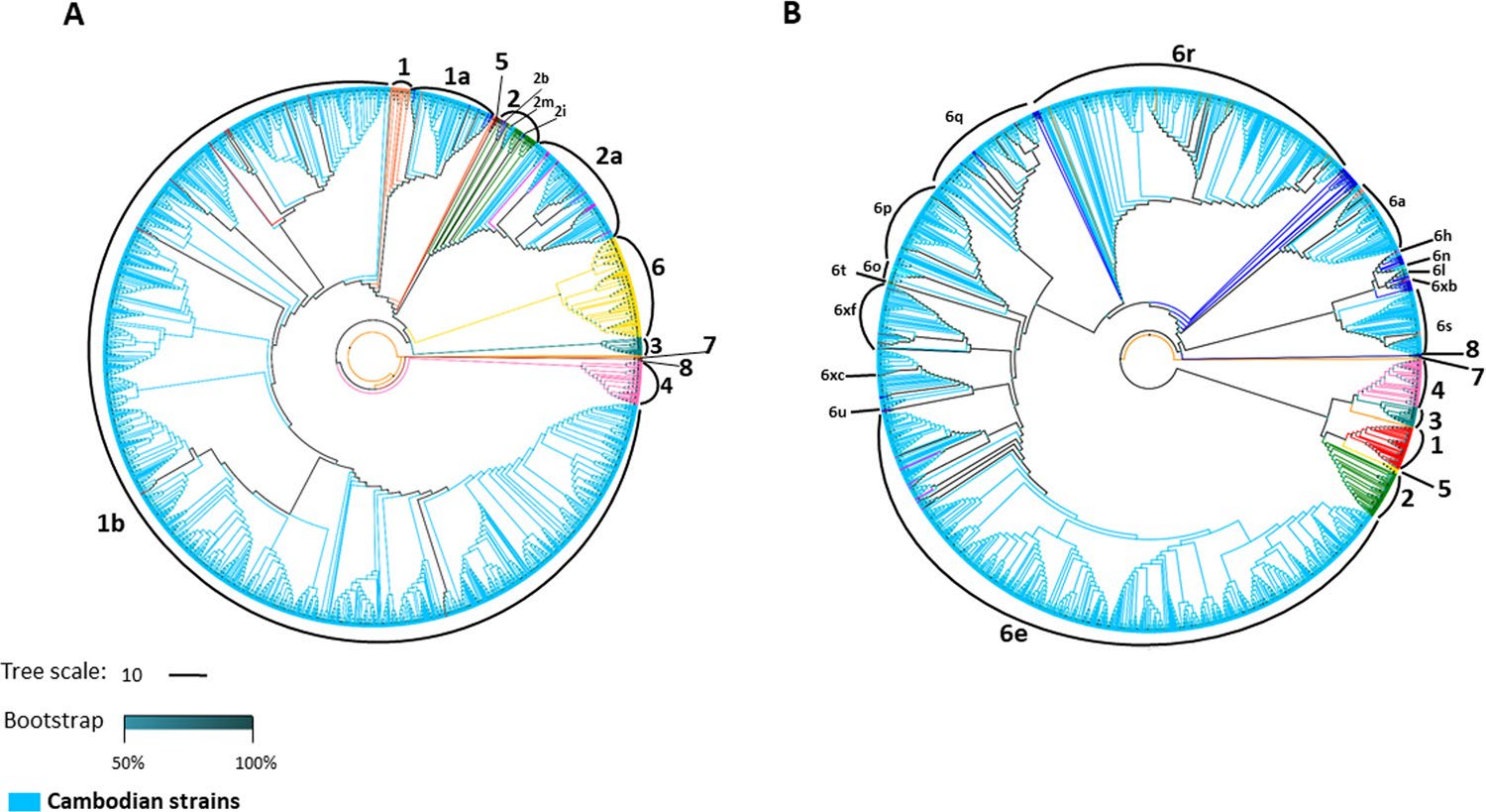
Phylogenetic trees of HCV NS5B sequences. Phylogenetic trees were inferred using the maximum likelihood (ML) method based on GTR + Γ + I (for HCV genotype none 6) and JC + Γ (for HCV genotype 6) models of nucleotide substitution with HCV genotype 8 as outgroup. (**A**) ML phylogenetic tree for HCV NS5B sequences from 1,691 Cambodian patients with HCV genotype none 6 (indicated in sky blue), and 285 GenBank reference sequences indicated in different colours (subtype 1a: orange; 1b: red; 2: green; 2a; magenta; 2b: purple; 2i: navy; 2m: golden red; 3: teal; 4: pink; 5: maroon; 6: yellow; 7: dark orange, and 8: dark purple). (**B**) ML phylogenetic tree for HCV NS5B sequences from 1,442 Cambodian patients with HCV genotype 6 (indicated in SkyBlue) and 285 GenBank reference sequences are indicated in different colours (genotype 1: red; 2: green; 3: teal; 4: pink; 5: yellow; 6a: salmon; 6e: magenta; 6h: medium purple; 6 l: dark slate grey 6n: turquoise; 6o: cyan; 6p: dark green; 6q: coral; 6r: dark golden red; 6s; rosy bran; 6t: olive; 6u: purple; 6xb: deep pink; 6xc: maroon; 6xf: brown; other subtypes within genotype 6 including 6b, 6c, and 6d, 6f, 6g, 6i, 6k, 6m, 6v, 6w, 6xa, 6xd, and 6xe: blue; 7: dark orange, and 8: dark blue).

Preliminary phylogenetic analysis performed on HCV non-structural 5B (NS5B) sequences obtained from 3,133 patients revealed the presence of HCV genotypes 1, 2, 6 and an unassigned genotype. HCV genotype 1 and 6 were the most predominant genotypes, found in 1,444 (46.1%) and 1,442 (46.0%) samples, respectively. A small number of samples were identified as genotype 2, accounting for 4.3% (134/3,133), followed by unclassified genotype (3.6%; 113/3,133). Subsequently, maximum likelihood (ML) phylogenetic trees of HCV genotype 6 and non-6 sequences including both genotype 1 and 2 were separately constructed. Figure 1 shows the phylogenetic patterns of HCV sequences within each genotype.

Among non-genotype 6 viruses, the viral sequences were well conserved. Among genotype 1, two viral subtypes were identified: 1b was the most predominant subtype with a frequency of 94% (n = 1,357) followed by a lower frequency of subtype 1a (6%; n = 87) (Fig. 1A). Within genotype 2 strains, 125 (93.3%) sequences were distributed into the 2a subtype cluster, 6 (4.5%) were 2 m subtype, 2 (1.5%) were 2b subtype, and only 1 sequence (0.7%) was 2i subtype.

A high genetic diversity was observed within the genotype 6 (Fig. 1B). The most common subtypes were 6e and 6r accounting for 43.5% (n = 628) and 22.9% (n = 331), respectively. Seventy (4.9%) sequences were not clustered with any known subtype of HCV genotype 6. The remaining sequences belong to subtypes: 6q 7.8% (n = 113), 6p 5.5% (n = 80), 6a 4.9% (n = 70), 6xf 4.4% (n = 63), 6s 4.2% (n = 61), 6o 0.7% (n = 10), 6 l 0.4% (n = 5), 6u 0.3% (n = 4), 6h 0.2% (n = 3), 6n 0.07% (n = 1), 6t 0.07% (n = 1), 6xb 0.07% (n = 1) and 6xc 0.07% (n = 1).

### Geographical distribution of HCV genotype

**Figure 2:**
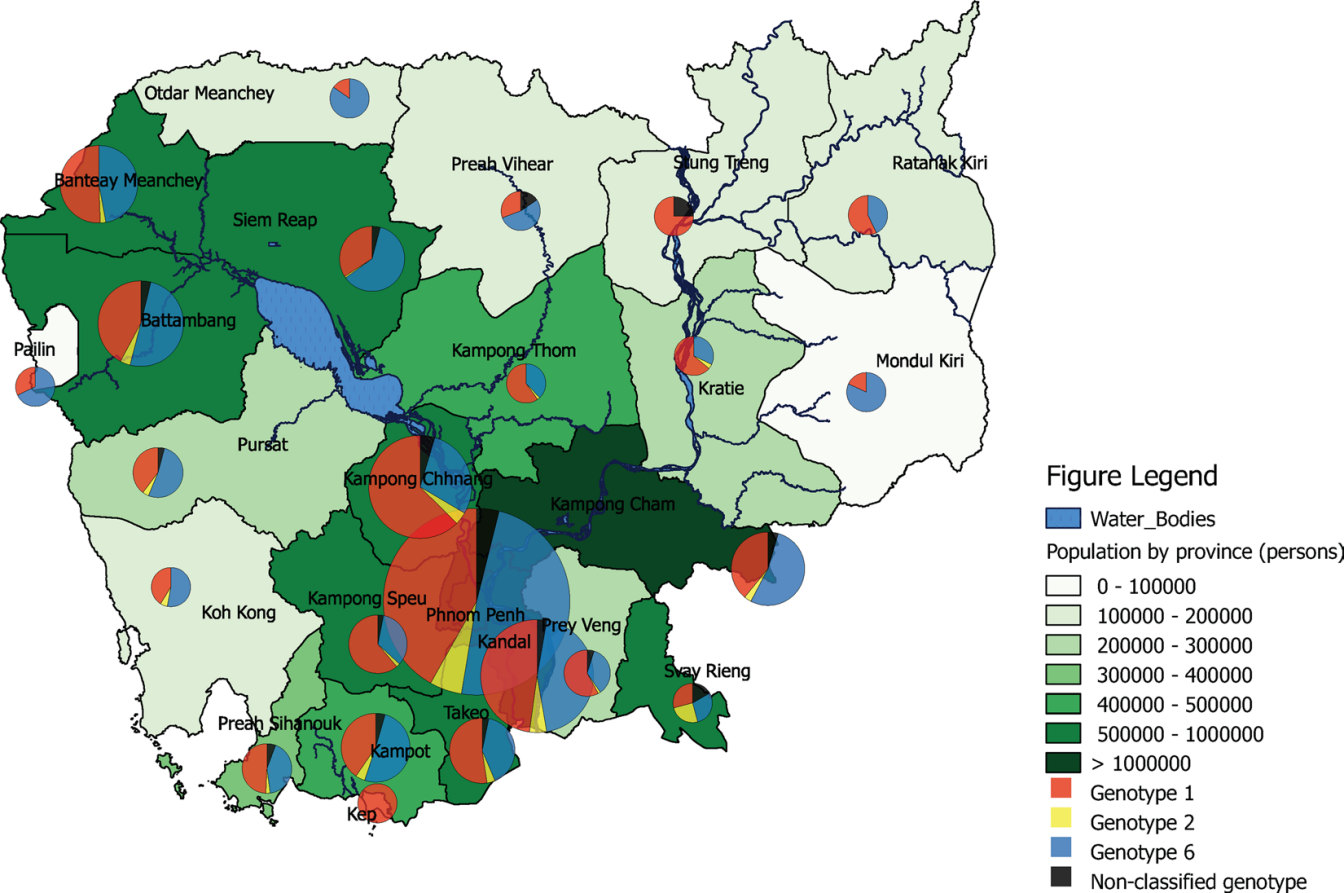
Geographical distribution of HCV genotypes by province in Cambodia. Different-sizes circles represent study population size of each province.

Patients included in the study were coming from all province across Cambodia. However, the number was unequal from one province to another. Figure 2 shows the distribution of HCV genotypes according to geographical area. When focusing on provinces with a high number of participants, we observed that the 3 viral genotypes detected in the present study was distributed similarly in these provinces, except Kampong Chhnang province where the proportion of HCV genotype 1 was much higher than other provinces (63% GT1 and 29% GT6 among n = 305 in Kampong Chhnang Province compared to 44% GT1 and 48% GT6 among n = 2,826 patients from all other provinces).

### HCV transmission risk factors and HCV genotypes

Patients were interviewed for risk factors of HCV infection including a history of invasive medical procedure, blood transfusion and drug use (Table 1). The majority of patients had a history of invasive medical procedures (64.7%). Having partners with known HCV infection and history of blood transfusion were also common (19.5% and 9.9%, respectively). The distribution of risk factors associated with HCV infection was comparable across genotypes.

### Clinical features of HCV infection and HCV genotypes

We found that the frequency of patients having a high HCV RNA viral load (VL) of greater than or equal to 800,000 IU/mL was highest amongst patients infected with HCV genotype 6 compared to other genotypes. In contrast, we observed that all patients shared similar clinical features, except for the fibroscan value. Patients infected with HCV genotype 1 had the highest level of liver stiffness as presented by a high value of fibroscan (median: 10.4 kPa) compared to those infected with HCV genotypes 2 (8.6 kPa), 6 (9.7 kPa), and unclassified genotype (9.2 kPa) (p < 0.05). Additionally, the distribution of fibrosis stage differed across genotypes: HCV GT1 had the greatest proportion of patients with fibrosis stage ≥ F3 (54%), followed by HCV genotype 6 (51%) and HCV genotype 2 with the smallest proportion (44%), although differences were not statistically significant (Table 1).

### HCV genotype and advanced fibrosis

To evaluate the association between HCV genotype and advanced fibrosis (defined as fibroscan value ≥ 20 kPa), we calculated the odds of having advanced fibrosis for 3,099 patients with fibroscan data. In univariate analysis, we found that HCV genotype 6 had lower odds of advanced fibrosis compared to HCV genotype 1 and this was consistently observed in the multivariable analysis after adjusting for age, sex, and body-mass-index (adjusted odds ratio (aOR) [95% confidence interval (CI)]: 0.7 [0.6–0.9], p < 0.01) (Table 2).

**Table 2:** Crude (cOR) and adjusted odds ratio (aOR) for advanced fibrosis of ≥20 kPa on Fibroscan (n = 3099).

Patient characteristics	cOR	95%CI	p-values	aOR	95%CI	p-values
**Genotype**						
1	1			1		
2	0.7	(0.5–1.1)	p = 0.15	0.7	(0.4–1.1)	p = 0.09
6	0.8	(0.7–0.9)	p < 0.01	0.7	(0.6–0.9)	p < 0.01
Non-classified	0.8	(0.5–1.3)	p = 0.31	0.8	(0.5–1.3)	p = 0.34
**Sex**						
Female	1			1		
Male	1.4	(1.1–1.6)	p < 0.001	1.4	(1.2–1.7)	p < 0.001
**Age, year**						
<40	1			1		
≥40 and <50	3.9	(2.0–7.5)	p < 0.001	4.0	(2.1–7.7)	p < 0.001
≥50 and <60	8.7	(4.7–16.2)	p < 0.001	8.9	(4.8–16.6)	p < 0.001
≥60 and <70	12.1	(6.5–22.5)	p < 0.001	12.7	(6.8–23.5)	p < 0.001
≥70	12.0	(6.2–23.2)	p < 0.001	13.1	(6.8–25.5)	p < 0.001
**Body-mass-index, kg/m** ^**2**^						
<20	1			1		
≥20 and <30	1.2	(0.9–1.5)	p = 0.13	1.3	(1.0–1.6)	p = 0.07
≥30	2.0	(1.3–3.0)	p < 0.001	2.1	(1.3–3.2)	p < 0.001

Notes: adjusted odds ratios are adjusted for sex, age (categorical <40; ≥40 and <50; ≥50 and <60; ≥60 and <70; ≥70 years old) and body-mass-index (<20; ≥20 and <30; ≥30 kg/m^2^).

## Discussion

Here, we report a large data of HCV molecular epidemiology in Cambodia based on demographic, clinical data and HCV sequences belonging to 3,133 patients presenting from the majority parts of Cambodia between September 2016 and December 2017. This study revealed a circulation of HCV genotype 1, 2, 6, and unclassified variants, with a predominance of genotypes 1 and 6, accounting for 46.1% and 46.0% of the study population, respectively. Our finding is in agreement with previous studies conducted in different populations and regions in Cambodia^15,16,18,20^, at least for the viral genotypes. However, it seems difficult to perform quantitative comparison for the proportions of each genotype reported in these studies as all previous studies were based on small population size, restricted geographical areas, and/or specific populations. For instance, HCV genotype 1 was found as the most prevalent strain (68%) in a study carried out on 28 HIV and HCV co-infected patients followed-up at Calmette Hospital in Phnom Penh, while HCV genotype 6 (25%) and genotype 2 (7%) were less frequent^16^. On the other hand, HCV genotype 6 was the most predominant among 25 HCV-infected immigrant workers from Cambodia in Thailand^18^ and among 11 HCV viremic adults living in Siem Reap province^20^. In a recent study, De Weggheleire *et al*. reported a predominance of both genotypes 1 (52.8%) and 6 (41.4%) among 87 HIV and HCV co-infected patients from Phnom Penh^15^.

When analyzing each genotype in-depth, we observed a low level of genetic diversity among HCV genotypes 1 and 2 present in Cambodia. Overall, the most predominant subtype was 1b and followed by small percentages of subtypes 2a, 1a, 2m, 2b, and 2i. The frequency of subtype 1b found in our study was comparable with previous report by De Weggheleire *et al*.^15^.

Within the genotype 6, there was a high genetic diversity with subtypes 6e as the most common and accounting for 43.5%. Subtype 6e is commonly detected in neighboring countries and it has been suggested that HCV subtype 6e originated from Viet Nam^23^. HCV subtype 6r was the second most common subtype found in 22.9% of HCV genotype 6 sequences. In contrast to the subtype 6e, it has been stated that HCV subtype 6r is specific to Cambodia, as it has been detected only in this country or among its native population living abroad^18,24^. Nevertheless, further molecular evolutionary analysis of this subtype is needed to confirm this hypothesis. Other subtypes representing low frequencies were also detected: 6q, 6p, 6a, 6xf, 6s, 6o, 6l, 6u, 6h, 6n, 6t, 6xb, 6xc, and unclassified genotype 6 variants. The great genetic diversity of HCV genotype 6 was also described in neighboring Laos^13^, Thailand^25^, and Vietnam^23^. This characteristic may indicate that HCV genotype 6 has circulated, adapted and evolved for a long period of time in Cambodia as previously suggested for other countries in the region^13,23^.

HCV genotype 3 circulates commonly among intravenous drug users through needle and syringe sharing^26^. Unsurprisingly, this genotype is completely absent in our study. This may be explained by a low number of intravenous drug users in the current study. As mentioned in Table 1, 18 out of 3,120 patients were drug users.

Among 3,133 HCV sequences included in the analysis, 113 (3.6%) remained unclassified, possibly related to new HCV variants. Subsequently, further investigation by analyzing other genes or full-length genome sequences of these viruses could be relevant.

All of the HCV genotypes detected were homogenously distributed in province where significant numbers of patients were included, except Kampong Chhnang province where the frequency of HCV genotype 1 is higher than other provinces. This observation suggests that there is no geographic specificity of the corresponding genotype. However, we were not able to explain the high frequency of HCV genotype 1 in Kampong Chnang province. It is important to note that our data was statistically biased and was not representative of the whole country, as patients included in the present study were self-refered to receive diagnosis and/or treatment for HCV in facility located in the Capital city.

Risk factors associated with HCV infection such as exposure to invasive medical procedures, blood transfusion, partner with HCV and a history of drug use were comparable for all HCV genotypes circulating in Cambodia. Among these factors, past experience of invasive medical procedures were reported by more than 60% of patients included in the present analysis and many cases are likely to be iatrogenic, since Cambodia has been known as a country with high rate of medical injections such as therapeutic injections and intravenous infusions^22,27,28^. Recently, we have reconstructed a likely transmission history of a massive iatrogenic HIV outbreak occurred in Roka (a rural commune in Cambodia) between 2014 and 2015. We showed that unsafe injections most likely led to this large outbreak which was also associated with a long-standing HCV transmission with multiple and independent sources of introduction^22^.

Very few studies have assessed natural history and clinical features of HCV genotype 6 compared to other genotypes. A cross-sectional study performed in 308 Southeast Asians in California, USA found no significant differences in virological and clinical characteristics between HCV genotype 6 and other genotypes^29^. A study conducted in Hong Kong, Seto *et al*. compared the natural history of 138 HCV genotype 1 patients (median age: 50 years old) with 78 HCV genotype 6 patients (median age: 46.5 years old) after a median follow-up period of over 5 years. In this survey, both genotypes had comparable liver biochemistry, HCV RNA viral load and similar rates of development of cirrhotic complications and mortality^30^. The findings of these studies suggested that viral genotype is not the main discriminating factor of disease outcome. In our study, it seems that HCV genotype 6 has higher viral load compared to other genotypes. However, we were not able to further explore the association between disease progression and viral genotypes, since our analysis was cross-sectional and many significant factors which impact the natural history of HCV such as age at the time of initial infection or duration of infection and host factors were not available.

In conclusion, we showed that molecular epidemiology of HCV in Cambodia is predominantly associated with two genotypes sharing similar proportions: genotypes 1 and 6. The most prevalent viral subtypes were 1b, followed by 6e and 6r. The route of transmission of HCV in Cambodia could be predominantly linked to invasive medical procedures including unsafe injection practices. This characteristic of epidemiology is specific to Cambodia. Further investigation is needed to better understand the evolution of HCV viral strain in Cambodia.

## Methods

### Study setting

In collaboration with the MoH of Cambodia, Médecins Sans Frontières (MSF– Doctors without borders) launched a HCV program inside the Hepato-Gastro Department of Preah Kossamak Hospital, a 254-bed national hospital in Phnom Penh, in September 2016 aimed at developing a simplified care model adapted for Cambodian context. MSF provided free testing and treatment services to patients seeking care either through the out-patient services or through MSF’s screening programs for at-risk patient groups, which include patients enrolled in HIV care, HIV patients who are female entertainment workers (FEW), transgender (TG) or men who have sex with men (MSM), and injection drug-users receiving needle-exchange and other support services from various collaborating non-governmental organizations. MSF operated two sites within the hospital: one dedicated for screening and diagnosis (Screening Site) and another for HCV treatment (Treatment Site).

### Study design

Between September, 2016 and December, 2017, 3,352 adult chronic HCV patients, 18 years of age or older, visiting HCV clinic of MSF were recruited to participate in the study. Among them, 3,133 patients gave their written consent to be included. MSF’s HCV service was open to any patients seeking diagnosis and/or treatment for HCV and consisted of patients from Phnom Penh and any of the other 23 provinces in Cambodia.

The current study was a prospective and anonymous analysis of data collected between September 19, 2016 and December 6, 2017 from HCV-infected adult patients who were enrolled in a cohort of the MSF’s clinics. The treatment regimen utilizes a combination of Sofosbuvir (NS5B inhibitor) and Daclatasvir (or Ledipasvir: NS5A inhibitor) for 12 weeks, as recommended by the current AASLD/IDSA HCV guideline^31^. REDCap electronic database (Research Electronic Data Capture; Vanderbilt University, USA)^32^, which is hosted at Epicentre, Médecins Sans Frontières (Paris, France), was used to store demographic (gender, age, place of residence etc.) and clinical data at the enrollment of all patients. Blood samples were collected for assessment of HCV infection.

All HCV-infected adults (≥18 years) whose demographic, clinical data and HCV sequences were available, were eligible in the present analysis.

### Ethics statement

All patients included in the present study provided written informed consent for the use of their demographic, clinical, and biological data. The study protocol was approved by the Cambodian National Ethics committee for Health Research. All methods were performed in accordance with relevant guidelines and regulations.

### Assessment of HCV infection and genotyping

All specimens tested positive for HCV antibodies by SD Bioline HCV (Standard Diagnostics, Inc., Rest-of-World regulatory version, Kyonggi-do, Korea) were assessed for HCV RNA viral load testing using the COBAS AmpliPrep/Cobas TaqMan HCV Quantitative Test, v2.0 platform (Roche) according to manufacturer’s instructions.

HCV genotype and subtype were determined for all samples with detectable HCV RNA viral load (>1.2 Log_10_ IU/mL) based on phylogenetic analysis of the HCV non-structural 5B (NS5B) genome region (371 bp) that was amplified using a semi-nested RT-PCR, as described previously^33^. The amplifications of the NS5B gene were performed at the Institut Pasteur du Cambodge (Phnom Penh, Cambodia). All PCR amplified fragments were sent for sequencing to a commercial sequencing facility (Macrogen, Inc., Seoul, South Korea) using the Big Dye Terminator v3.1 Cycle Sequencing kit (Applied Biosystems). Chromatograms were sent back electronically to the Institut Pasteur in Cambodia for verification by visual inspection using CEQ 2000 (Beckman Coulter) software. Viral sequences were aligned with reference sequences for HCV subtypes available in GenBank database (Supplementary Table 1). Phylogenetic trees were constructed using the maximum likelihood (ML) method based on GTR + Γ + I^34^ (for HCV genotype none 6) and JC + Γ^35^ (for HCV genotype 6) models of nucleotide substitution, as recommended by the Find Best DNA/Protein models program inserted in the MEGA7 software^36^.

### Statistical analysis

For the descriptive analysis of patients in the cohort, mean, standard deviation (SD) and ANOVA were used to describe normally distributed variables, and median, inter-quartile-range (IQR) and Kruskal-Wallis test were used to describe non-normally distributed continuous variables. Proportions and chi-squared tests were used to describe categorical variables. To compare virological and clinical features of different HCV genotypes, HCV RNA viral load at baseline, liver stiffness by transient elastography using Fibroscan, transaminase values, and comorbidities of all patients were analyzed. To examine whether HCV genotypes were associated with advanced fibrosis (≥20 kPa), univariate and multivariate logistic regression analysis was performed using the following variables: gender, age (<40; ≥40 and <50; ≥50 and <60; ≥60 and <70; ≥70 years old) and body-mass-index (<20; ≥20 and <30; ≥30 kg/m^2^) with HCV genotype 1 as the reference group.

All statistical tests were performed two-sided at alpha 0.05 using STATA version 13.1 software (STATACorp LP, College Station, Texas, USA, 2016).

### Geographic HCV mapping

During the initial assessment of HCV infection, the province of residence was collected for each patient. Mapping was performed by importing Cambodian shapefiles from Open Development Cambodia for the Economic Census of Cambodia 2011 (Ministry of Planning, National Institute of Statistics) and genotype data from this study into QGIS version 2.16.0 (Development Team - Open Source Geospatial Foundation Project, 2016)^37^.

### Nucleotide sequence accession number

All HCV NS5B sequences included in the present study were submitted to GenBank and registered under accession numbers: MK436248 - MK436360 (unassigned genotype), MK436361 - MK437938 (genotype non-6), and MK437939 - MK439380 (HCV genotype 6).

## Supplementary information

Supplementary Table 1

## Data Availability

Epidemiological and clinical datasets analyzed during the current study are available from MSF but terms and conditions apply to the availability of these data which are not publicly available. Data are however available from the authors (M.I.) upon reasonable request and with permission of the ethical committee and MSF. Accession numbers of HCV reference sequences used in the current analysis are available in Supplementary Materials.

## References

[1] World Health Organisation. *Global hepatitis report, 2017*, ISBN 978-992-4156545-4156545 (2017).

[2] Wang H (2013). Eight novel hepatitis C virus genomes reveal the changing taxonomic structure of genotype 6. The Journal of general virology.

[3] Smith DB (2014). Expanded classification of hepatitis C virus into 7 genotypes and 67 subtypes: updated criteria and genotype assignment web resource. Hepatology.

[4] Borgia SM (2018). Identification of a Novel Hepatitis C Virus Genotype From Punjab, India: Expanding Classification of Hepatitis C Virus Into 8 Genotypes. The Journal of infectious diseases.

[5] Smith, D. B. *et al*. International Committee on Taxonomy of Viruses (ICTV). HCV Classification. A web resource to manage the classification and genotype and subtype assignments of hepatitis C virus. at, https://talk.ictvonline.org/ictv_wikis/flaviviridae/w/sg_flavi/56/hcv-classification (2017).

[6] Gower E, Estes C, Blach S, Razavi-Shearer K, Razavi H (2014). Global epidemiology and genotype distribution of the hepatitis C virus infection. Journal of hepatology.

[7] Polaris Observatory HCVC (2017). Global prevalence and genotype distribution of hepatitis C virus infection in 2015: a modelling study. The lancet. Gastroenterology & hepatology.

[8] Markov PV (2009). Phylogeography and molecular epidemiology of hepatitis C virus genotype 2 in Africa. The Journal of general virology.

[9] Shenge JA, Odaibo GN, Olaleye DO (2019). Phylogenetic analysis of hepatitis C virus among HIV/HCV co-infected patients in Nigeria. PloS one.

[10] Shenge JA, Odaibo GN, Olaleye DO (2018). Genetic Diversity of Hepatitis C Virus Among Blood Donors and Patients with Clinical Hepatitis in Ibadan, Nigeria. Archives of basic and applied medicine.

[11] Narahari S, Juwle A, Basak S, Saranath D (2009). Prevalence and geographic distribution of Hepatitis C Virus genotypes in Indian patient cohort. Infection, genetics and evolution: journal of molecular epidemiology and evolutionary genetics in infectious diseases.

[12] Gededzha MP, Selabe SG, Blackard JT, Kyaw T, Mphahlele MJ (2014). Near full-length genome analysis of HCV genotype 5 strains from South Africa. Infection, genetics and evolution: journal of molecular epidemiology and evolutionary genetics in infectious diseases.

[13] Pybus OG (2009). Genetic history of hepatitis C virus in East Asia. Journal of virology.

[14] Ol HS (2009). Prevalence of hepatitis B and hepatitis C virus infections in potential blood donors in rural Cambodia. The Southeast Asian journal of tropical medicine and public health.

[15] De Weggheleire A (2017). A cross-sectional study of hepatitis C among people living with HIV in Cambodia: Prevalence, risk factors, and potential for targeted screening. PloS one.

[16] Lerolle N (2012). High Frequency of Advanced Hepatic Disease among HIV/HCV Co-Infected Patients in Cambodia: The HEPACAM Study (ANRS 12267). J AIDS Clinic Res.

[17] Martinello M, Amin J, Matthews GV, Dore GJ (2016). Prevalence and Disease Burden of HCV Coinfection in HIV Cohorts in the Asia Pacific Region: A Systematic Review and Meta-Analysis. AIDS reviews.

[18] Akkarathamrongsin S (2011). Seroprevalence and genotype of hepatitis C virus among immigrant workers from Cambodia and Myanmar in Thailand. Intervirology.

[19] Fujimoto M (2018). A seroepidemiological survey of the effect of hepatitis B vaccine and hepatitis B and C virus infections among elementary school students in Siem Reap province, Cambodia. Hepatology research: the official journal of the Japan Society of Hepatology.

[20] Yamada H (2015). Seroprevalence, genotypic distribution and potential risk factors of hepatitis B and C virus infections among adults in Siem Reap, Cambodia. Hepatology research: the official journal of the Japan Society of Hepatology.

[21] Sarmati L (2003). Infection with human herpesvirus-8 and its correlation with hepatitis B virus and hepatitis C virus markers among rural populations in Cambodia. The American journal of tropical medicine and hygiene.

[22] Rouet F (2018). Massive Iatrogenic Outbreak of Human Immunodeficiency Virus Type 1 in Rural Cambodia, 2014-2015. Clinical infectious diseases: an official publication of the Infectious Diseases Society of America.

[23] Li C (2014). The genetic diversity and evolutionary history of hepatitis C virus in Vietnam. Virology.

[24] Li C, Lu L, Zhang X, Murphy D (2009). Entire genome sequences of two new HCV subtypes, 6r and 6s, and characterization of unique HVR1 variation patterns within genotype 6. Journal of viral hepatitis.

[25] Wasitthankasem R (2015). Genotypic distribution of hepatitis C virus in Thailand and Southeast Asia. PloS one.

[26] Verachai V (2002). Prevalence and genotypes of hepatitis C virus infection among drug addicts and blood donors in Thailand. The Southeast Asian journal of tropical medicine and public health.

[27] Goyet S (2014). Risk factors for hepatitis C transmission in HIV patients, Hepacam study, ANRS 12267 Cambodia. AIDS and behavior.

[28] Vong S (2005). Rapid assessment of injection practices in Cambodia, 2002. BMC public health.

[29] Nguyen NH (2010). Risk factors, genotype 6 prevalence, and clinical characteristics of chronic hepatitis C in Southeast Asian Americans. Hepatology international.

[30] Seto WK (2010). Natural history of chronic hepatitis C: genotype 1 versus genotype 6. Journal of hepatology.

[31] Panel AIHGH (2015). C guidance: AASLD-IDSA recommendations for testing, managing, and treating adults infected with hepatitis C virus. Hepatology.

[32] Harris PA (2009). Research electronic data capture (REDCap)–a metadata-driven methodology and workflow process for providing translational research informatics support. Journal of biomedical informatics.

[33] Budkowska A (2011). Synonymous mutations in the core gene are linked to unusual serological profile in hepatitis C virus infection. PloS one.

[34] Nei, M. & Kumar, S. Molecular Evolution and Phylogenetics. *Oxford University Press, New York* (2000).

[35] Jukes, T. H. & Cantor, C. R. Evolution of protein molecules. *In Munro HN, editor, Mammalian Protein Metabolism* Academic Press, New York, 21–132 (1969).

[36] Kumar S, Stecher G, Tamura K (2016). MEGA7: Molecular Evolutionary Genetics Analysis Version 7.0 for Bigger Datasets. Molecular biology and evolution.

[37] Cambodia National Institute of Statistics. *Economic Census of Cambodia*, https://www.nis.gov.kh/index.php/en/about/general-information/12-publications/16-economic-census-2011-final-results (2011).

