# Supplementary material for: Molecular epidemiology of hepatitis C virus in Cambodia during 2016–2017
Source: Sci Rep. 2019 May 13;9:7314. doi: 10.1038/s41598-019-43785-4 (PMC6514207; doi:10.1038/s41598-019-43785-4)
Supplement: Supplementary file 1 — Supplementary Table 1 [file 41598_2019_43785_MOESM1_ESM.docx]

**Molecular epidemiology of hepatitis C virus in Cambodia during 2016 - 2017**

Janin Nouhin^1*^, Momoko Iwamoto^2,3^, Sophearot Prak^1^, Jean-Philippe Dousset^3^, Kerya Phon^4^, Seiha Heng^4^, Alexandra Kerleguer^4^, Mickaël Le Paih^3^, Philippe Dussart^1^, David Maman^2^, François Rouet^1, †^

^1^ Virology Unit, Institut Pasteur du Cambodge, Institut Pasteur International Network, Phnom Penh, Cambodia

^2^ Epicentre, Paris, France

^3^ Médecins Sans Frontières – France, Phnom Penh, Cambodia

^4^ Medical Laboratory, Institut Pasteur du Cambodge, Institut Pasteur International Network, Phnom Penh, Cambodia

^†^deceased

***Corresponding author**

Janin Nouhin, Ph.D., Virology Unit, Institut Pasteur du Cambodge, Institut Pasteur International Network, Phnom Penh, Cambodia

Present address: Stanford University School of Medicine, Division of Infectious Diseases and Geographic Medicine,

1000 Welch Road, Suite 202, Palo Alto, CA 94304, USA

Tel: +1(650)-398-5135

E-mail: [jnouhin@stanford.edu](mailto:jnouhin@stanford.edu)

^†^deceased

**Supplementary Table 1.** HCV reference sequence dataset (n=285) used in phylogenetic analysis

| **HCV Genotype** | **No.** | **Genbank accession number** |
| --- | --- | --- |
| 1 | 7 | HQ537007, AJ851228, KC248195, KJ439780, KJ439779, KJ439776, KJ439777 |
| 1a | 7 | EF032886, AF009606, NC004102, M62321, M67463, HQ850279, EF407457 |
| 1b | 10 | AB189092, AF165056, LC210214, LC109122, AM494937, EU482881 |
|  |  | D90208, M58335, EU781827, EU781828 |
| 1c | 4 | KC844047, D14853, AY051292, AY651061 |
| 1d | 1 | KJ439768 |
| 1e | 1 | KC248194 |
| 1g | 1 | AM910652 |
| 1h | 2 | KC248198, KC248199 |
| 1i | 1 | KJ439772 |
| 1j | 1 | KJ439773 |
| 1k | 1 | KJ439774 |
| 1l | 3 | KC248193, KC248197, KC248196 |
| 1m | 2 | KJ439778, KJ439782 |
| 1n | 2 | KJ439781, KJ439775 |
| 2 | 8 | JF735116, JF735118, JF735117, JF735119, JF735110, KC197236, KC197237, KC197239 |
| 2/6 | 2 | DQ155502, DQ155559 |
| 2a | 11 | JF722466, KX346812, KF676351, JX677168, AY746460, GQ206089, AF238482, AB047645, D00944, AB047639, HQ639944 |
| Continued |  |  |
| **HCV Genotype** | **No.** | **GenBank accession number** |
| 2b | 6 | KC844048, KC197226, D10988, AB030907, AB661388, AB661382 |
| 2c | 4 | KC197227, JX227950, D50409, JX227949 |
| 2d | 1 | JF735114 |
| 2e | 1 | JF735120 |
| 2f | 2 | KC844042, KC844050 |
| 2i | 2 | KC197229, DQ155561 |
| 2j | 3 | HM777358, JF735113, HM777359 |
| 2k | 4 | KC197234, JX227952, AB031663, JX227953 |
| 2l | 2 | AY257465, GU054449 |
| 2m | 2 | JX227967, JF735111 |
| 2q | 2 | FN666428, FN666429 |
| 2r | 1 | JF735115 |
| 2t | 1 | KC197238 |
| 2u | 1 | JF735112 |
| 3 | 1 | JF735124 |
| 3a | 6 | DQ437509, GQ356206, D17763, D28917, X76918, JN714194 |
| 3b | 3 | KC441467, D49374, JQ065709 |
| 3d | 1 | KJ470619 |
| 3e | 1 | KJ470618 |
| 3g | 2 | JX227954, JF735123 |
| Countinued |  |  |
| **HCV Genotype** | **No.** | **GenBank accession number** |
| 3h | 2 | JF735126, JF735121 |
| 3i | 2 | FJ407092, JX227955 |
| 3k | 2 | JF735122, D63821 |
| 4 | 10 | FJ025854, JX227964, JF735127, JF735132, JF735131, JF735130, JF735129, JF735138, JF735135, JF735134 |
| 4a | 4 | Y11604, AB795432, DQ988074, DQ418789 |
| 4b | 1 | FJ462435 |
| 4c | 1 | FJ462436 |
| 4d | 4 | DQ516083, DQ418786, FJ462437, EU392172 |
| 4f | 3 | EF589161, EU392175, EU392174 |
| 4g | 3 | FJ462432, JX227971, JX227963 |
| 4k | 3 | EU392173, FJ462438, EU392171 |
| 4l | 2 | FJ839870, JX227957 |
| 4m | 2 | FJ462433, JX227972 |
| 4n | 2 | FJ462441, JX227970 |
| 4o | 2 | FJ462440, JX227977 |
| 4p | 1 | FJ462431 |
| 4q | 1 | FJ462434 |
| 4r | 2 | FJ462439, JX227976 |
| 4s | 1 | JF735136 |
| 4t | 1 | FJ839869 |
| Continued |  |  |
| **HCV Genotype** | **No.** | **GenBank accession number** |
| 4v | 4 | JX227960, HQ537009, JX227959, HQ537008 |
| 4w | 2 | FJ025855, FJ025856 |
| 5 | 1 | KT595242 |
| 5a | 4 | KC844046, EUH1480, AF064490, Y13184 |
| 6 | 28 | EF116158, AY754618, AY754627, AY754633, AY754637, AY754615, DQ278891, DQ278893, JX183558, JX183553, JX183554, JX183551, JX183549, JX183550, KJ470620, KJ470621, KJ470622, KJ470623, KJ470624, KJ470625, KC844039, KC844040, KJ567652, KJ567650, KJ567649, KJ567648, KJ567644, HE580419 |
| 6a | 6 | HQ912955, AY859526, HM031216, EU246930, Y12083, HQ639936 |
| 6b | 2 | D84262, GU049397 |
| 6c | 2 | EF424629, KP324209 |
| 6d | 1 | D84263 |
| 6e | 6 | DQ314805, JX103137, KM252779, KM252780, EU246932, EU246931 |
| 6f | 4 | DQ835760, DQ835764, EU246936, GU049373 |
| 6g | 2 | DQ314806, D63822 |
| 6h | 3 | HM009308, KF437714, D84265 |
| 6i | 2 | DQ835770, DQ835762 |
| 6j | 2 | DQ835769, DQ835761 |
| 6k | 1 | AY878651 |
| 6l | 2 | EF424628, JX183556 |
| 6m | 2 | DQ835767, DQ835766 |
| Continued |  |  |
| **HCV Genotype** | **No.** | **GenBank accession number** |
| 6n | 4 | DQ278894, DQ835768, KP324281, EU246938 |
| 6o | 3 | KJ470360, EF424627, EU246934 |
| 6p | 2 | AY894532, EF424626 |
| 6q | 3 | EF424625, EF116182, EF116191 |
| 6r | 10 | EU408328, EF116173, EF116153, EF116154, EF116163, EF116174, EF116184, EF116186, EF116199, KM252786, |
| 6s | 4 | EU408329, EF116169, EF116185, AY434110 |
| 6t | 2 | EF632071, EU246939 |
| 6u | 1 | EU246940 |
| 6v | 3 | EU798761, EU798760, EU158186 |
| 6w | 3 | DQ278892, EU643834, EU643836 |
| 6xa | 3 | EU408330, EU408331, EU408332 |
| 6xb | 2 | JX183552, KJ567645 |
| 6xc | 1 | KJ567651 |
| 6xd | 3 | KM252789, KM252790, KM252791 |
| 6xe | 2 | JX183557, KM252792 |
| 6xf | 4 | KT190711, KT190710, KJ567646, KJ567647 |
| 7a | 1 | EF108306 |
| 7b | 1 | KX092342 |
| 8 | 3 | MH590698, MH590699, MH590700, MH590701 |
